# Design of Cr-Based Molecular Electrocatalyst Systems for the CO_2_ Reduction Reaction

**DOI:** 10.1021/acs.accounts.4c00283

**Published:** 2024-08-06

**Authors:** Megan E. Moberg, Charles W. Machan

**Affiliations:** Department of Chemistry, University of Virginia, PO Box 400319, Charlottesville, Virginia 22904-4319, United States

## Abstract

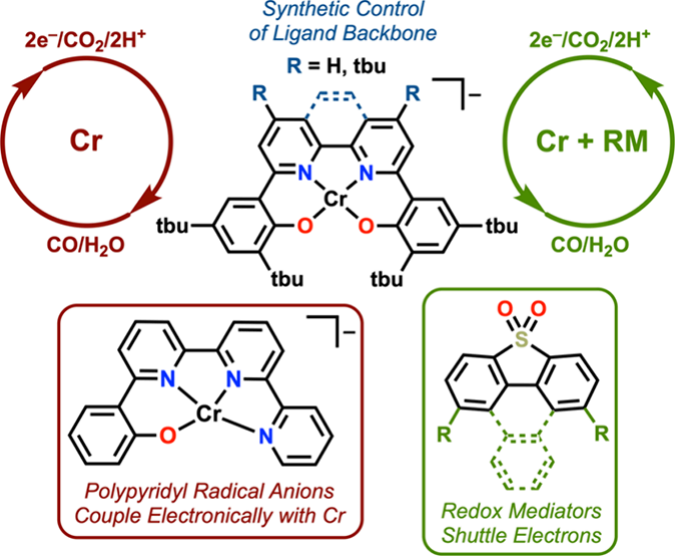

Human influence on the climate system was recently summarized by the sixth Intergovernmental Panel on Climate Change (IPCC) Assessment Report, which noted that global surface temperatures have increased more rapidly in the last 50 years than in any other 50-year period in the last 2000 years. Elevated global surface temperatures have had detrimental impacts, including more frequent and intense extreme weather patterns like flooding, wildfires, and droughts. In order to limit greenhouse gas emissions, various climate change policies, like emissions trading schemes and carbon taxes, have been implemented in many countries. The most prevalent anthropogenic greenhouse gas emitted is carbon dioxide (CO_2_), which accounted for 80% of all U.S. greenhouse gas emissions in 2022. The reduction of CO_2_ through the use of homogeneous electrocatalysts generally follows a two-electron/two-proton pathway to produce either carbon monoxide (CO) with water (H_2_O) as a coproduct or formic acid (HCOOH). These reduced carbon species are relevant to industrial applications: the Fischer–Tropsch process uses CO and H_2_ to produce fuels and commodity chemicals, while HCOOH is an energy dense carrier for fuel cells and useful synthetic reagent. Electrochemically reducing CO_2_ to value-added products is a potential way to address its steadily increasing atmospheric concentrations while supplanting the use of nonrenewable petrochemical reserves through the generation of new carbon-based resources. The selective electrochemical reduction of CO_2_ (CO_2_RR) by homogeneous catalyst systems was initially achieved with late (and sometimes costly) transition metal active sites, leading the field to conclude that transition metal complexes based on metals earlier in the periodic table, like chromium (Cr), were nonprivileged for the CO_2_RR. However, metals early in the table have sufficient reducing power to mediate the CO_2_RR and therefore could be selective in the correct coordination environment. This *Account* describes our efforts to develop and optimize novel Cr-based CO_2_RR catalyst systems through redox-active ligand modification strategies and the use of redox mediators (RMs). RMs are redox-active molecules which can participate cocatalytically during an electrochemical reaction, transferring electrons—often accompanied by protons—to a catalytic active site. Through mechanistic and computational work, we have found that ligand-based redox activity is key to controlling the intrinsic selectivity of these Cr compounds for CO_2_ activation. Ligand-based redox activity is also essential for developing cocatalytic systems, since it enables through-space interactions with reduced RMs containing redox-active planar aromatic groups, allowing charge transfer to occur within the catalyst assembly. Following a summary of our work, we offer a perspective on the possibilities for future development of catalytic and cocatalytic systems with early transition metals for small molecule activation.

## Key References

- HooeS. L.; DresselJ. M.; DickieD. A.; MachanC. W.Highly Efficient Electrocatalytic Reduction of CO_2_ to CO by a Molecular Chromium Complex. ACS Catal.2020, 10 ( (2), ), 1146–1151.^1^*First Cr-based homogeneous electrocatalyst for selective CO_2_ reduction with a proposed catalytic cycle where C–OH bond cleavage is the rate-determining step en route to carbon monoxide formation.*
- ReidA. G.; MorenoJ. J.; HooeS. L.; BaughK. R.; ThomasI. H.; DickieD. A.; MachanC. W.Inverse Potential Scaling in Co-Electrocatalytic Activity for CO_2_ Reduction through Redox Mediator Tuning and Catalyst Design. Chem. Sci.2022, 13 ( (33), ), 9595–9606.36091894 10.1039/d2sc03258aPMC9400620(2)*Two Cr-based electrocatalysts with different intrinsic activity were examined with four different redox mediators to determine how structural changes on catalyst and mediator affect coelectrocatalysis.*
- ReidA. G.; HooeS. L.; MorenoJ. J.; DickieD. A.; MachanC. W.Homogeneous Electrocatalytic Reduction of CO_2_ by a CrN_3_O Complex: Electronic Coupling with a Redox-Active Terpyridine Fragment Favors Selectivity for CO. Inorg. Chem.2022, 61 ( (43), ), 16963–16970.36260749 10.1021/acs.inorgchem.2c02013(3)*Redox-active bipyridine ligand frameworks were previously used in Cr-based molecular electrocatalysts. In studies with a terpyridine-based ligand framework, it was found that carbon dioxide reduction still occurred; however, the catalyst needed to be reduced by an additional electron equivalent*.
- HooeS. L.; MorenoJ. J.; ReidA. G.; CookE. N.; MachanC. W.Mediated Inner-Sphere Electron Transfer Induces Homogeneous Reduction of CO_2_ via Through-Space Electronic Conjugation. Angew. Chem. Int. Ed.2022, 61 ( (1), ), e202109645.10.1002/anie.20210964534695281(4)*First example of coelectrocatalytic carbon dioxide reduction reliant on an inner-sphere mechanism through the combination of a Cr-based electrocatalyst with an aromatic sulfone-based redox mediator, where the key cocatalyst species assembled in solution following CO_2_ binding.*

## Introduction

The amount of recorded electricity consumption was 14 times greater in 2022 (4.07 trillion kWh) than in 1950 and global energy consumption continues to increase.^5,6^ The corresponding need for energy generation has proliferated large-scale processes which emit CO_2_ as a waste product. Atmospheric concentrations of CO_2_ have risen steadily from a preindustrial (ca. 1750) level of 280 ppm to over 420 ppm. As an effective absorber of electromagnetic radiation in spectral regions which do not overlap with other gaseous molecules, CO_2_ contributes to increased energy capture from solar radiation, termed the “greenhouse effect”. Since a key source of anthropogenic CO_2_ emissions is fossil fuel combustion,^7,8^ there is an interest in shifting to renewable energy sources. The inherent fluxionality of renewable electricity (e.g., diurnal availability of solar energy) could be mitigated by storage in chemical bonds, enabling redesignation of CO_2_ from waste to energy carrier, and potentially displacing the use of nonrenewable petrochemicals for carbon-based feedstocks.^9,10^ Indeed, the electrochemical reduction of CO_2_ to produce important chemicals, would be a sustainable alternative to current approaches for producing fossil fuels. Although desirable, processes which mediate the electrochemical reduction of CO_2_ to highly reduced products like methane via an 8H^+^/8e^–^ reduction pathway can be energy intensive and nonselective.^11^

Because of the challenges associated with the distribution of active sites in heterogeneous materials, homogeneous catalysts can offer an advantage in selectivity. For example, 2H^+^/2e^–^ reduction pathways to form CO (and water as a coproduct) or formic acid are achieved with high selectivity by homogeneous electrocatalysts.^11^ Both CO and formic acid are relevant to industrial applications; the former can be used with H_2_ in the Fischer–Tropsch process to produce commodity chemicals and fuels,^12^ while the latter is useful synthetically and in fuel cells.^13^

Since these electrochemical reactions are proton dependent, a point of control is the modulation of proton donor activity with respect to the basicity of key intermediates.^14^ Although modification of the overpotential for an electrochemical reaction is a natural consequence of altered proton donor activity, there is a benefit to achieving concerted electron and proton transfer steps, since these can be used to avoid stepwise reduction and protonation, which otherwise might limit the reaction kinetically or introduce thermodynamic penalties.^15^ Homogeneous catalyst systems are generally explored in organic solvent systems as a practical consequence of their limited solubility in aqueous conditions, allowing for the use of very weak acids which do not dissociate in solution, disfavoring the thermodynamically favorable hydrogen evolution reaction (HER).^15^ Successful design of catalyst systems must therefore balance the proton and electron inventory required during the optimization of a reaction pathway, rendering the manner of electron delivery an additional point of possible control.

Electrocatalysts for the CO_2_RR have been synthesized with 3*d*, 4*d*, and 5*d* metal centers,^16^ however, the current state-of-the-art homogeneous catalyst is a trimethylanilinium (TMA)-modified Fe(III) *meso*-tetraphenylporphyrin [Fe(o-TMA)]^5+^,^17^ the base framework ([FeTPP]^+^) of which has been studied since 1988.^18^ Mechanistic studies have enabled advancements in catalytic performance, both through tuning reaction conditions and modifying the ligand framework.^19−21^ For instance, pendent hydroxy (−OH) moieties, were introduced to the base catalyst by Costentin et al. in 2012 ([Fe((OH)_8_TPP)]^+^).^20^ The −OH moieties improved CO_2_ binding through hydrogen bonding, which also accelerated subsequent rate-determining bond cleavage, significantly enhancing catalysis.^20^ Later work by Azcarate et al. showed that through-bond inductive substituent effects could stabilize the initial Fe^0^–CO_2_ adduct, leading to catalytic enhancement.^22^ In parallel, Azcarate et al. also disclosed [Fe(o-TMA)]^5+^, where the inclusion of cationic trimethylanilinium groups resulted in an electron-withdrawing effect that lowered the catalytic operating potential.^17^ In conjunction with this effect, the positively charged trimethylanilinium groups induced a strong Coulombic effect on the Fe^0^–CO_2_ adduct; the combination of these two effects resulted in the most active homogeneous molecular catalyst for the CO_2_RR reported to date.^17,23^

**Figure 1:**
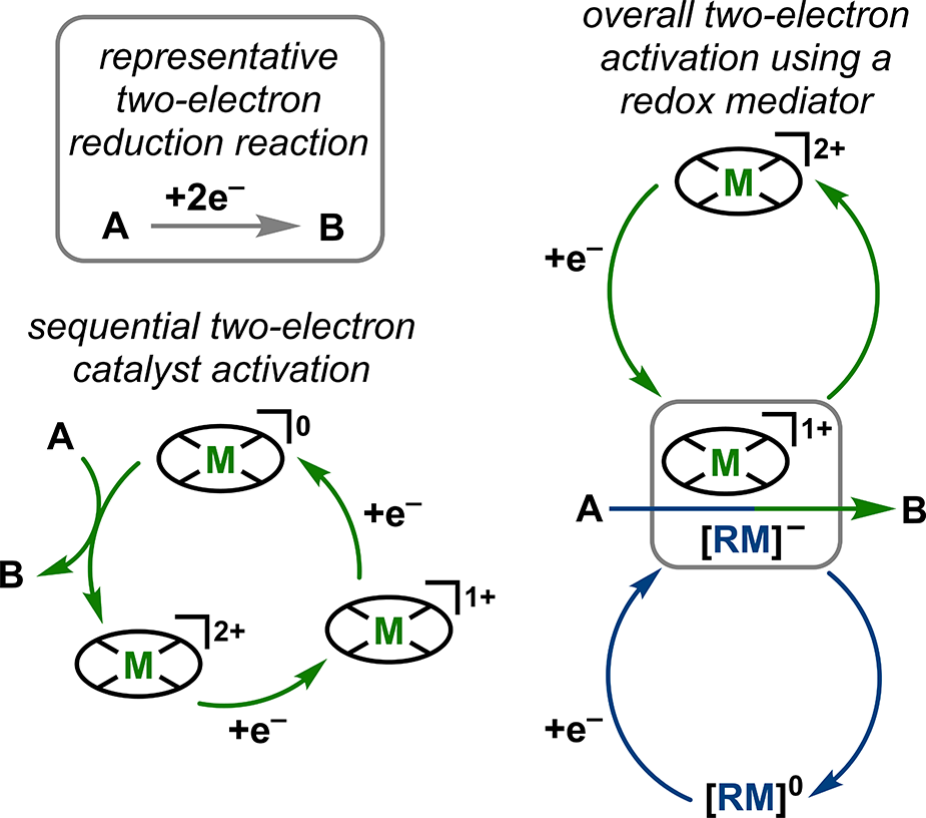
Generic schemes illustrating the effect of a RM on the catalytic mechanism of a two-electron reduction reaction.

An alternative way to improve homogeneous molecular catalysis without altering the structure of the catalyst is with a redox mediator (RM) as a part of a cocatalytic system (Figure 1).^24^ RMs transfer electrons to active sites, accessing alternative pathways. RMs are common in biological systems, for example, ubiquinone assists in shuttling electrons and protons in the electron transport chain during mitochondrial respiration.^25^ In 2020, Smith et al. disclosed the first use of a series of nicotinamide adenine dinucleotide (NADH) RMs for the CO_2_RR using the aforementioned [Fe(TPP)]^+^ catalyst, leading to a 13-fold enhancement in activity.^26^ These studies exemplify the value of mechanistically guided iterative synthetic design in improving catalyst performance.

**Figure 2:**
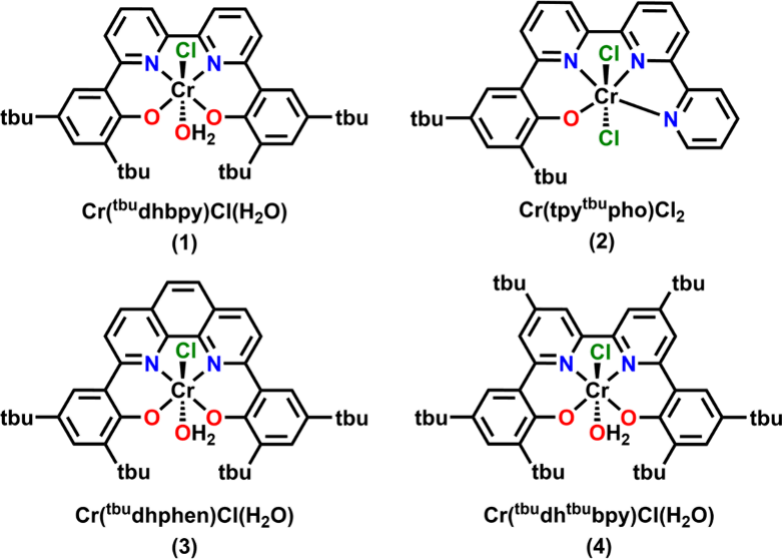
Structures of Cr complexes discussed in this *Account*.

Although stoichiometric reactivity between the Cr/Mo/W triad and CO_2_ was known, until recently little success in CO_2_RR had been achieved.^27^ An initial example was the work of Kubiak and co-workers, who showed that zerovalent Mo and W tetracarbonyl cores were effective precatalysts upon the inclusion of redox-active bipyridine (bpy) ligands.^28^ It was determined that catalytic activation required a two-electron reduction of the parent zero-valent tetracarbonyl compounds prior to CO release to create a vacant site for catalysis, increasing the thermodynamic and kinetic penalties associated with the CO_2_RR.^27,28^ Considerable success with later transition metals led to an unofficial consensus that Cr/Mo/W were nonprivileged for the CO_2_RR.^15,27^ Our group has since developed a series of Cr-based catalysts that show electrocatalytic activity for the selective reduction CO_2_ to CO (Figure 2). The key to achieve high activity and selectivity at low overpotentials was the interaction of electrons with opposite spin located on the Cr center and the redox noninnocent bpy backbone.^29^ To manage electron transfer, we have also developed RMs which coordinate to the Cr center, improving activity. In this *Account*, we summarize the results obtained from iterative catalyst design based on a mechanistic understanding of essential inner- and outer-coordination sphere components of these reactions. We conclude with a discussion on the role of RMs in mechanism and activity.

## Homogeneous Cr-Based Electrocatalysts for Protic CO_2_RR

**Figure 3:**
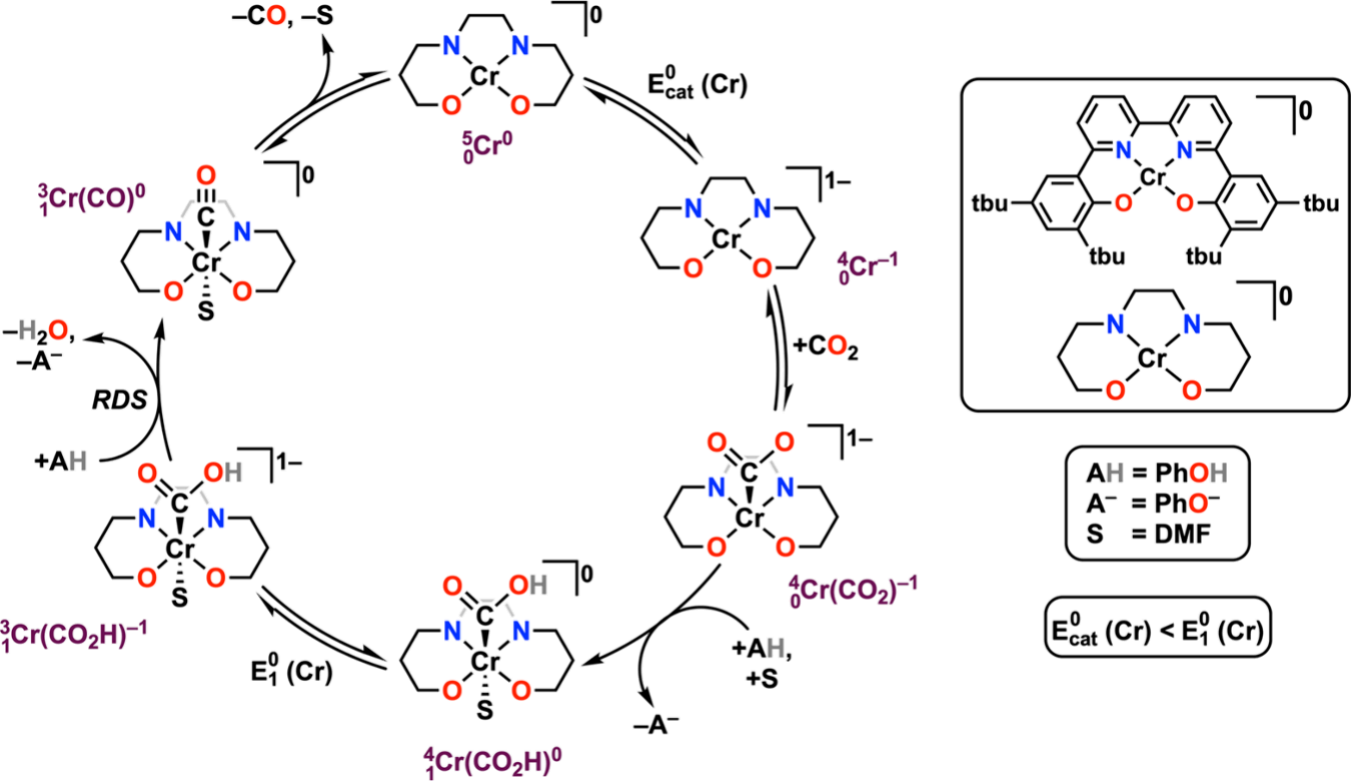
Proposed catalytic mechanism for the CO_2_RR mediated by Cr complexes with a bipyridine-based dianionic ligand framework from mechanistic and computational studies.

The first reported quantitatively selective Cr-based electrocatalyst for the CO_2_RR was Cr(^tbu^dhbpy)Cl(H_2_O) (**1**), where the ligand precursor, (^tbu^dhbpy(H)_2_), is 6,6′-di(3,5-di-*tert*-butyl-2-hydroxybenzene)-2,2′-bipyridine (Figure 2).^1^ Cyclic voltammetry (CV) experiments demonstrated that only upon addition of the weak acid phenol (PhOH, p*K*_a_(DMF) = 18.8)^30^ as a proton source under CO_2_ saturation could an electrocatalytic response for the CO_2_RR be observed. Catalysis originated from a reversible reduction feature with an *E*_1/2_ = −1.95 V vs Fc^+^/Fc in the absence of substrate. Controlled potential electrolysis (CPE) was used to determine the reaction products, Faradaic efficiency (FE, ratio of electrons consumed to product detected), and long-term stability of the catalytic response. At an applied potential of −2.1 V vs Fc^+^/Fc, a FE_CO_ = 96 ± 8% was observed (H_2_ amount unquantifiable) over the course of 15.0 catalyst turnovers. Mechanistic studies revealed a catalytic rate law with first-order concentration dependencies on catalyst, CO_2_, and PhOH, as well as established that the catalyst was reduced by two electrons before becoming active. The turnover frequency (TOF) of the system was estimated to be 5.7 ± 0.1 s^–1^ using CV methods. The empirically derived rate expression and TOF were used to evaluate experimental data through digital CV simulations, which were consistent with an *ECEC′* mechanism, where *E* corresponds to a reversible electron transfer step and *C* refers to an irreversible chemical step. In the *ECEC′* framework, the proposed mechanism began with the reduction of a neutral Cr species to a monoanionic one that binds CO_2_ (Figure 3). Since CO_2_ binding is unfavorable, the ensuing irreversible protonation of the anionic Cr–CO_2_ adduct to a neutral Cr–CO_2_H complex represents the first chemical step of the reaction. Subsequent one-electron reduction generates a monoanionic Cr–CO_2_H species, at which point rate-determining proton-assisted cleavage of the C–OH bond to generate a Cr carbonyl species with a water coproduct can occur.

Following this initial publication, a mechanistic DFT study evaluated this mechanistic proposal and assessed the underlying contributors to the observed selectivity and activity (Figure 3).^29^ Consistent with experiment, reduction of a neutral Cr species  to a monoanionic Cr species  was identified as relevant to CO_2_ binding. In this notation, minima are represented as , reflecting the invariant coordination of the [^tbu^dhbpy]^2–^ ligand. The transition from an S = 2 to S = 3/2 state during reduction of the neutral Cr species  to a monoanionic Cr species  reflects an important electronic structure change. The  species is best described as a high-spin Cr(II) system, with four unpaired electrons localized on Cr. Reduction to  places an electron on the redox-active bipyridine ligand with an opposite spin orientation to the high-spin Cr(II) center, resulting in a Cr(II)(bpy^•–^) configuration. This distribution of added electrons—including the antiferromagnetic pairing of an electron on the ligand with those formally on the Cr center—is crucial for the observed selectivity for CO_2_.

**Figure 4:**
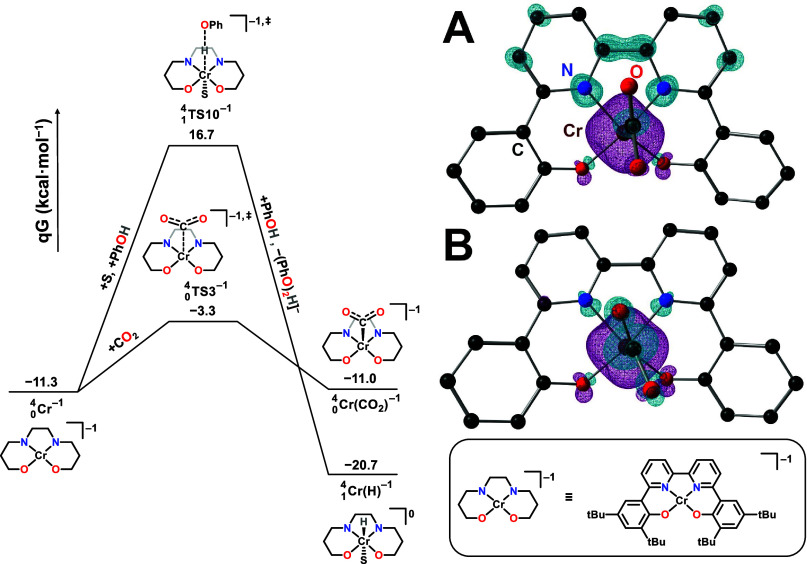
Free energy profile of the CO_2_ binding versus PhOH protonation of the active catalyst species . (**A**) spin density for the CO_2_ binding transition state and (**B**) spin density in the CO_2_ adduct .

Comparing the thermodynamic and kinetic parameters for CO_2_ binding to  to generate , a Cr(III)(bpy^0^) species, to those corresponding to protonation by PhOH to obtain a chromium hydride  shows that the former reaction is slightly endergonic, but ∼20 kcal/mol more kinetically accessible (Figure 4). Although the formation of a  is thermodynamically favored, the required restructuring of electron density to the Cr center to achieve formal proton transfer introduces a significant kinetic barrier. Analysis of the frontier Kohn–Sham orbitals and spin density in the transition state for CO_2_ binding (Figure 4A) suggests that the incipient bond between Cr and CO_2_ results from pairing the bpy-based electron with a Cr-based one. This aligns with the lower reaction barrier since this combination of electrons would be antiferromagnetically paired and therefore capable of forming a bond without a kinetically limiting change in spin configuration. Further, the relevant antibonding orbital of CO_2_ has significant π acid character,^29^ which matches a more diffuse molecular orbital distributed between Cr and the bipyridine fragment. These observations are consistent with work on Re and Mn complexes containing bpy ligands, where kinetic selectivity benefits for CO_2_ over protonation are observed through spin-pairing between the metal center and the reduced ligand framework.^31−34^

Subsequent protonation and reduction of the Cr(III)(bpy^0^) species  generates the proposed resting state of the catalytic cycle,  (Figure 3). This triplet configuration is best described as a high-spin Cr(III) center (S = 3/2) antiferromagnetically paired with a bpy-based radical ion, Cr(III)(bpy^•–^), reflecting the ligand field strength of the hydroxycarbonyl. In the transition state for the second proton transfer step from PhOH to , the added electron density on the bpy fragment is again important, delocalizing into the Cr–CO π-backbonding interaction that forms during C–OH bond cleavage to obtain the  product. Analysis of electronic and structural parameters of these species suggests that the bond cleavage is concerted with the proton transfer reaction. CO loss from the Cr(II) species  to close the catalytic cycle by reforming  reveals a minimal barrier, consistent with experimental evidence showing no interaction with CO by CV.^1^ This is also consistent with the open-shell nature of all proposed Cr intermediates, which can be attributed to the relatively weak ligand field imparted by [^tbu^dhbpy]^2–^, as well as the absence of formally Cr(0) species during catalysis thanks to the redox activity of the bpy fragment. Analyzing the complete catalytic cycle via the energetic span method of Kozuch indicated that the C–OH bond cleavage step contributed 72% to the observed TOF, while the remaining contribution came from CO_2_ binding.^35^ Therefore, the C–OH bond cleavage step could be designated as the turnover frequency determining transition state (TDTS) for the bpy-based catalytic cycle, as was originally proposed from mechanistic studies.

## Expanding the Redox Activity of the Ligand Framework

In light of the proposed participation of the ligand backbone, we were inspired by the work of Chang and co-workers on terpyridine-based ligand frameworks to examine how the expansion of ligand redox activity alters catalysis.^36^ A terpyridine(tpy)-based ligand with an [N_3_O]^−^ coordination environment was prepared, Cr(tpy^tbu^pho)Cl_2_ (Figure 2).^3^ Unlike the bpy-based compound, the tpy-based one must be reduced by three electron equivalents to become activated at a reversible reduction feature (*E*_1/2_ = −2.18 V vs Fc^+^/Fc) 230 mV more negative than the bpy-based complex. CPE experiments performed at −2.3 V vs Fc^+^/Fc with Cr(tpy^tbu^pho)Cl_2_ demonstrated a FE_CO_ = 93 ± 7% with 2.66 ± 0.05% H_2_ detected. The turnover frequency (TOF_CPE_) derived from this electrolysis experiment is 1.82 s^–1^, which is less than the TOF of 7.12 s^–1^ obtained for the bpy-based system during a comparable experiment.

**Figure 5:**
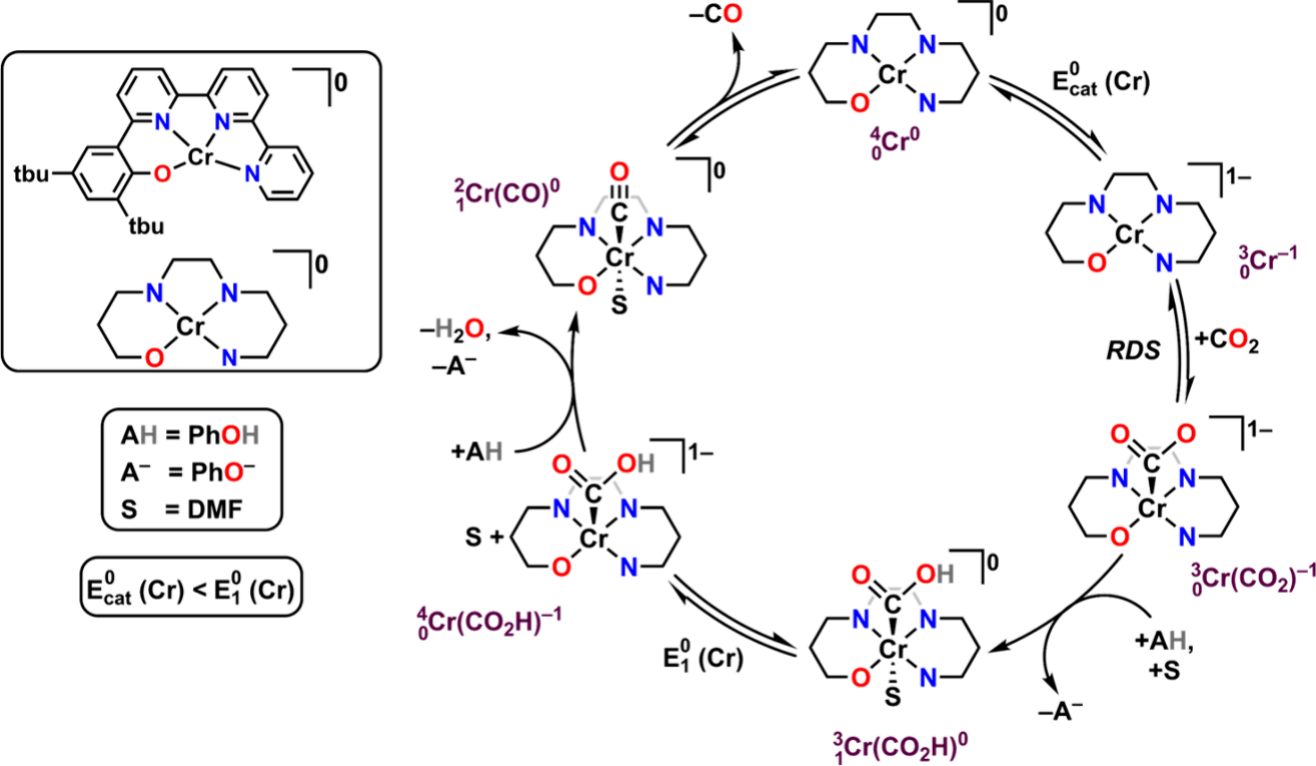
Proposed catalytic mechanism for CO_2_RR mediated by Cr complexes with a terpyridine-based ligand framework from mechanistic and computational studies.

Although the tpy-based complex still reduces CO_2_ to CO with near quantitative selectivity, the rate-determining step shifts to CO_2_ binding (Figure 5). Mechanistic studies showed saturation of the electrocatalytic current with minimal concentrations of PhOH added to solution and the catalytic rate expression was dependent only on the concentration of the Cr complex and CO_2_. Computational analyses by DFT methods indicated that these changes could be attributed to the simultaneous decrease in intrinsic ligand charge (L_3_X vs L_2_X_2_) and increase in ligand redox activity. In the four-coordinate monoanionic active state of the tpy-based complex,  (Figure 5) a triplet diradical in the ligand is antiferromagnetically paired with a high-spin Cr(II) center, Cr(II)(tpy^••2–^). Contrary to the bpy-based complex, the electrons which form the Cr–C bond during CO_2_ binding by the tpy-based  complex are localized on the metal with significant σ character, resulting in a heightened barrier for CO_2_ binding. This change in electronic structure also renders CO_2_ binding as slightly exergonic overall, likely due to a greater resemblance of the four-coordinate monoanion to the electronic structure of the product CO_2_ adduct. The consequence of greater Cr participation is also observed in a decreased barrier to Cr protonation: the difference in transition state energies between CO_2_ binding to form  and protonation to generate a hydride decreases to approximately 8 kcal/mol.

Stepwise protonation and reduction of  produces the second key tpy-based diradical state in the five-coordinate species which precedes C–OH bond cleavage  (Figure 5). Although an S = 1/2 configuration resembling Cr(III)(tpy^••2–^) is slightly lower in energy upon axial DMF coordination, the expanded ligand redox activity of tpy renders the higher-spin five-coordinate  species energetically accessible (Δ*G* = +0.2 kcal/mol upon DMF loss). The  species has the lowest energy transition state for C–OH bond cleavage and is best described as a part of a continuum between Cr(III)(tpy^••2–^) and Cr(II)(tpy^•–^) configurations. Overall, the increased redox-activity of tpy assists in significantly diminishing the barrier of this chemical reaction step relative to the bpy-based compound, since two ligand-based electron equivalents are coupled to Cr and are available for transfer, rather than one. Although the favorability of C–OH bond cleavage in  to generate the six-coordinate  is enhanced (DMF recoordinates), the increased barrier for CO_2_ binding to  slows the overall rate. These results suggest that to leverage the benefits of expanded redox activity of the tpy-based ligand framework in the future, the needs for CO_2_ binding and C–OH bond cleavage must be balanced.

## Effect of Bpy-Based Ligand Modifications

**Figure 6:**
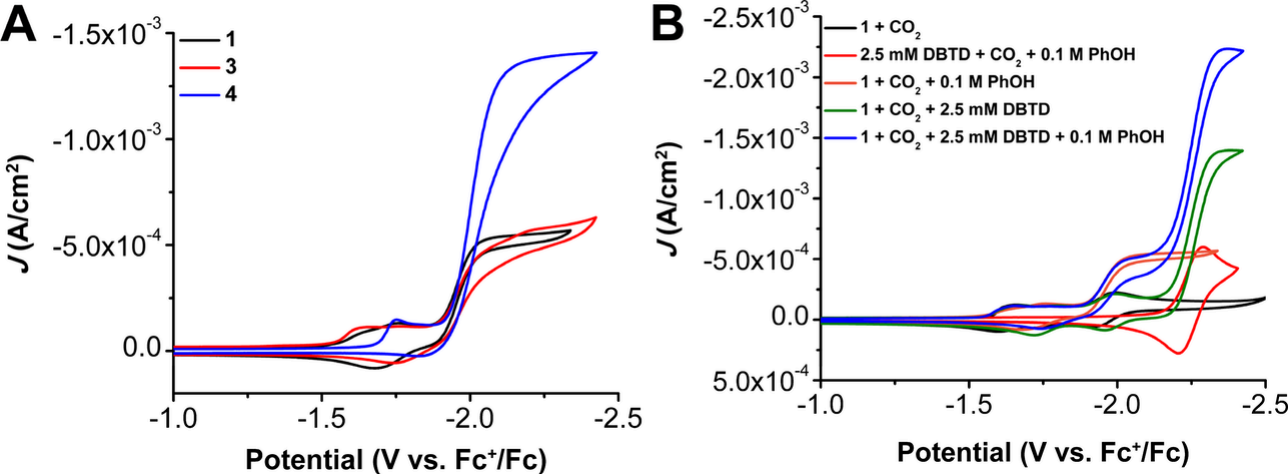
(**A**) CVs comparing Cr(^tbu^dhbpy)Cl(H_2_O) **1**, Cr(^tbu^dhphen)Cl(H_2_O) **3**, and Cr(^tbu^dh^tbu^bpy)Cl(H_2_O) **4** under CO_2_ saturation. (**B**) CVs comparing 2.5 mM DBTD with Cr(^tbu^dhbpy)Cl(H_2_O) **1**. Conditions: 1 mM catalyst, 0.1 M PhOH, 0.1 M TBAPF_6_/DMF; glassy carbon disc working electrode, glassy carbon rod counter electrode, Ag/AgCl pseudoreference; referenced to internal Fc^+^/Fc; 100 mV/s scan rate.

Parallel efforts have explored modifying the bpy framework to tune catalytic activity. One example is the change of the ligand backbone to phenanthroline (phen), Cr(^tbu^dhphen)Cl(H_2_O) (**3**), while the other added electron-donating groups on the bpy ligand, Cr(^tbu^dh^tbu^bpy)Cl(H_2_O) (**4**) (Figure 2).^2,37^ The three redox events observed for **3** are more negative than of **1** by 10 mV. CPE studies on **3** showed quantitative FE_CO_ = 101 ± 3% with a TOF = 4.90 s^–1^. Although the activity of **3** is slightly less than **1**, mechanistic DFT studies suggested that the two complexes were approximately isoergic with respect to the TDTS (C–OH bond cleavage, ΔΔ*G*^‡^ = 0.2 kcal/mol). Minimal differences between complexes **1** and **3** are also predicted for CO_2_ binding by their corresponding four-coordinate monoanions, which can both be described as Cr(II) with a radical anion localized on the bpy or phen fragment, respectively. A comparison of the transition states for CO_2_ binding by **1** and **3**, reveals that the participating KS orbitals are analogous, with significant π* bpy or phen character that contributes to the formation of the chromium–carbon bond as described above. The relatively minor electronic differences are a consequence of the lack of participation by the additional ring in the phen backbone (Figure 6).

CV experiments with **4** revealed a shift to more negative catalytic potentials relative to the other catalysts due to the electron-donating quality of the *tert*-butyl groups on bpy; catalysis originates at a redox feature with an *E*_1/2_ = −2.00 V vs Fc^+^/Fc, which is 50 mV more negative than **1**) (Figure 6). During CPE studies with PhOH and CO_2_ saturation, **4** showed quantitative FE_CO_ with a TOF of 9.29 s^–1^. Consistent with the expected scaling relationship, where a more negative potential correlates to greater activity for analogous reactions,^38^ mechanistic DFT studies predicted that barrier for the TDTS C–OH bond cleavage mediated by **4** (whose active species generated at a more negative potential) is approximately 1 kcal/mol lower in energy than those of **1** and **3**.^2^

## Redox Mediators and Co-Electrocatalysis

Electrocatalytic processes like the CO_2_RR require the overall transfer of multiple electrons and protons. When this occurs at a single active site, high-energy intermediates can be required, since it is often difficult to add multiple electron equivalents to a metal center at the same potential, especially with first-row transition metals that prefer open-shell configurations. RMs that transfer electron equivalents to the active site can circumvent the need for high-energy intermediates since they distribute the required redox balance between multiple species (Figure 1).^24^

**Figure 7:**
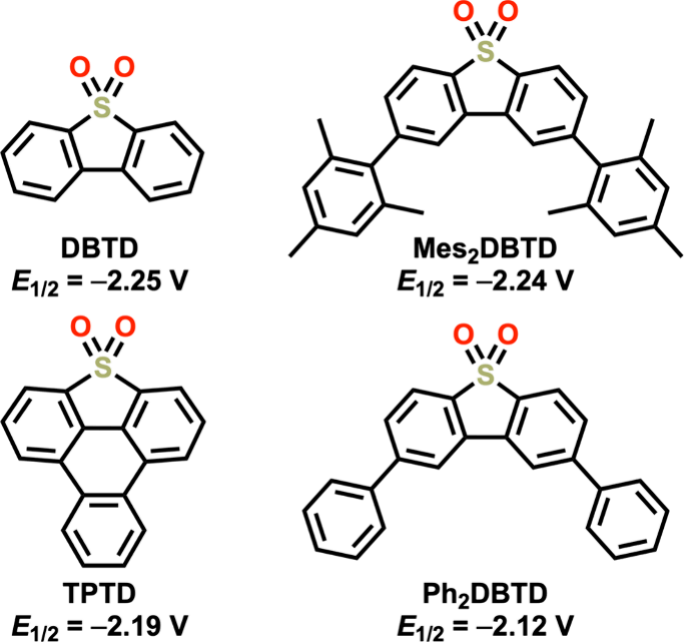
Structures of the dibenzothiophene-5,5-dioxide (DBTD), triphenylothiophene-4,4-dioxide (TPTD), 2,8-dimesityldibenzothiophene-5,5-dioxide (Mes_2_DBTD), and 2,8-diphenyldibenzothiophene-5,5-dioxide (Ph_2_DBTD) RMs; potentials versus Fc^+^/Fc.

Because of its well-defined electrochemical properties at reducing potentials, dibenzothiophene-5,5-dioxide (DBTD) (Figure 7) was initially chosen as a RM since the reduction potential is more negative than the Cr complexes described above. In dye-sensitized solar cells or bioelectrocatalysis–similar to the energy gradients used during biological electron transfer–going from more reducing standard reduction potentials to less reducing can help to direct the flow of electrons to specific active sites.^24^ Creating a gradient of redox potentials to the active site provides a thermodynamic driving force for the reaction in the forward direction and disfavors back electron transfer.

The addition of DBTD to a solution of **1** under Ar saturation conditions indicates no interaction occurs at the DBTD^0/–^ reduction (−2.25 V vs Fc^+^/Fc), which is approximately 0.3 V more negative than the catalytic reduction potential of the Cr complex.^4^ However, under CO_2_ saturation, a large irreversible increase in current occurs at the DBTD^0/–^ redox couple consistent with coelectrocatalytic activity (Figure 6B). No reaction was observed with CO_2_ for either **1** or DBTD in this potential range in the absence of a proton donor, based on which it was concluded that a catalyst species with emergent properties was forming when the Cr complex and RM were combined. Indirect experimental evidence of this was observed in a proportional shift of *E*_cat/2_ for the cocatalytic feature when the concentration of DBTD was increased relative to **1**. Further, including decamethylcobaltocenium, which lacks the ability to coordinate to Cr, to the reaction mixture does not result in cocatalytic activity under these aprotic conditions. A CPE experiment with **1** and DBTD at −2.30 V vs Fc^+^/Fc showed CO production with 91 ± 10% FE at a TOF of 36.8 s^–1^; the carbonate coproduct to CO obtained as the result of the reductive disproportionation of two equivalents of CO_2_ was verified by ^13^C NMR.

**Figure 8:**
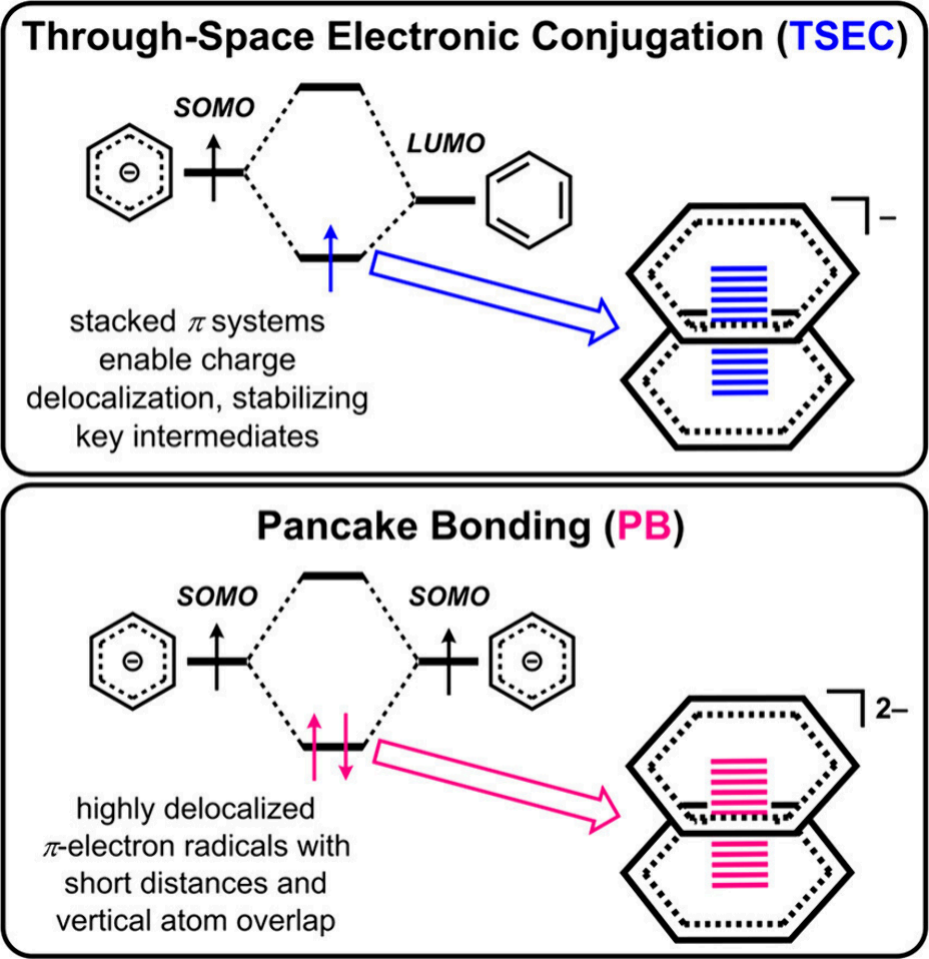
Stabilizing forces for key intermediates in the inner-sphere cocatalytic mechanisms described here. Reproduced from ref (2). with permission from the Royal Society of Chemistry. Available under a CC-BY-NC license. Copyright 2022 Reid, A.G.; Moreno, J.J.; Hooe, S.L.; Baugh, K.R.; Thomas, I.H.; Dickie, D.A.; Machan, C.W.

Although sulfones are weak ligands, it was speculated that a dative covalent interaction with the Cr center was occurring when DBTD was reduced. Using mechanistic and computational studies, an inner-sphere catalytic mechanism was proposed where **1** undergoes an overall two-electron reduction to form  as described above, Cr(II)(bpy^•–^), followed by kinetically accessible but thermodynamically unfavorable sequential head-to-tail binding of two equivalents of CO_2_ to Cr generating , a Cr(III)(bpy^0^) species. The doublet monoanion [DBTD]^−^ can axially coordinate to the  complex in a reaction which is thermodynamically favorable and triggers irreversible C–O bond cleavage to produce CO and CO_3_^2–^. The interaction occurs through the combination of through-space electronic conjugation (TSEC; charge transfer involving a single electron between stacked π systems, Figure 8),^4^ weak bonding between DBTD and Cr, and dispersion interactions. The TSEC results from the aromatic framework of the doublet monoanion [DBTD]^−^ interacting with the neutral bpy ligand of .

When PhOH is added to **1** and DBTD under CO_2_ saturation, the CV trace reflects the presence of two electrocatalytic responses: the intrinsic response of **1** at −2.10 V vs Fc^+^/Fc, followed by a second S-shaped wave triggered by DBTD reduction at −2.25 V vs Fc^+^/Fc (Figure 6B). Relative to the intrinsic performance of **1**, under cocatalytic conditions a CPE experiment at −2.30 V vs Fc^+^/Fc revealed that under these protic reaction conditions CO was produced with 102 ± 14% FE at a TOF of 65.3 s^–1^, a 9-fold increase over the performance of **1** without the RM. The reduced [DBTD]^−^ RM was proposed to be intercepting the resting state of the intrinsic catalytic cycle , a Cr(III)(bpy^•–^) configuration, displacing an equivalent of DMF solvent to create an overreduced cocatalyst assembly  through an equilibrium reaction. This interaction relies on the formation of a Cr–O bond with the RM and dispersion interactions, and the type of TSEC shifts to pancake bonding (PB), where a pair of electrons is shared across aromatic groups (Figure 8).^2^ In the proposed PB, the aromatic portion of the doublet monoanion [DBTD]^−^ pairs with the bpy-based radical anion of the  complex. With DBTD, this equilibrium displacement reaction was predicted to be only slightly favorable by DFT, which suggested that further improvements could be made by optimizing PB through synthetic changes to the RM and Cr complex.

In a PB bonding interaction, achieving vertical atom overlap at short distances is important to create a pathway for electron transfer.^2^ Therefore, it was reasoned that altering the DBTD core to shift its reduction potential positive and add aromatic character would increase cocatalytic activity. In total, three new RMs were synthesized (TPTD, Mes_2_DBTD, and Ph_2_DBTD) (Figure 7), all of which show a reversible one-electron redox feature under Ar saturation conditions.^2^ The *E*_1/2_ values of these RMs are more positive than DBTD: Ph_2_DBTD *E*_1/2_ = −2.12 V, TPTD *E*_1/2_ = −2.19 V, and Mes_2_DBTD *E*_1/2_ = −2.24 V vs Fc^+^/Fc. CPE experiments performed on complexes **1** and **4** with these new RMs and DBTD uniformly exhibit selective CO production. Interestingly, with either **1** or **4** as a catalyst, the observed coelectrocatalytic TOF_CPE_ was ordered identically with respect to RM: TPTD > Ph_2_DBTD > DBTD > Mes_2_DBTD. It was noteworthy that the two RMs with the most positive reduction potentials (TPTD and Ph_2_DBTD) demonstrated the greatest cocatalytic activity, an inverse potential scaling effect. Although Mes_2_DBTD and DBTD have similar reduction potentials, the increased steric profile of the mesityl functional groups presented a kinetic barrier for the formation of the cocatalyst assembly with Mes_2_DBTD, lowering its relative activity. Comparing TPTD, Ph_2_DBTD, and DBTD, the observation of inverse potential scaling for cocatalytic activity is evidence of an inner-sphere mechanism, since an outer-sphere mechanism would be expected to conform to Marcus Theory, where greater potential differences correspond to greater rates.

**Figure 9:**
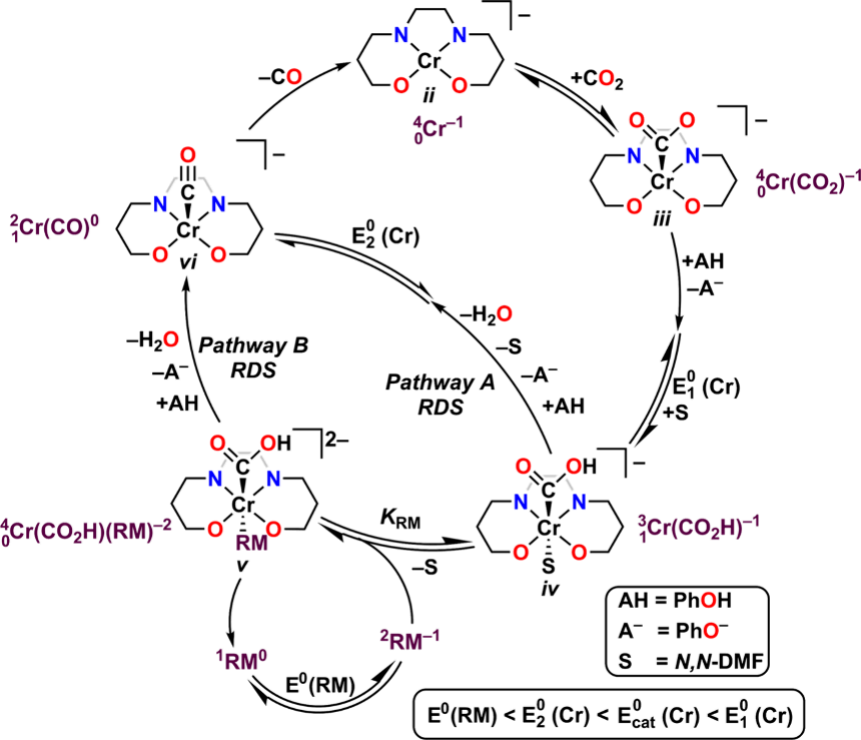
Proposed catalytic mechanism for CO_2_RR mediated by Cr complexes with a bipyridine-based tetradentate dianionic ligand framework from mechanistic and computational studies.

The intrinsic catalytic performance of the Cr complexes proceeds through *Pathway A* as the rate-determining step (RDS) in Figure 9, whereas the coelectrocatalytic cycle proceeds through *Pathway B* as the RDS. The equilibrium displacement reaction wherein the reduced RM binds to species *iii* to form species *iv* with accompanying DMF loss controls the observed activity: increasing the equilibrium binding constant of the RM, *K*_RM_, increases the observed TOF. The inverse scaling phenomenon can be attributed to the redox potential of the RM serving as an approximation of its frontier orbital energy. Since the PB interactions rely in part on vertical atom–atom overlap between the π-frameworks of the reduced bpy-backbone and the reduced RM, interaction strength should increase as the relevant orbitals become closer in energy (Figure 8). Thus, more positive RM reduction potentials reflect bringing the orbital energy levels of RM and Cr complex closer together, increasing the observed cocatalytic rate by favoring the formation of  relative to .

**Figure 10:**
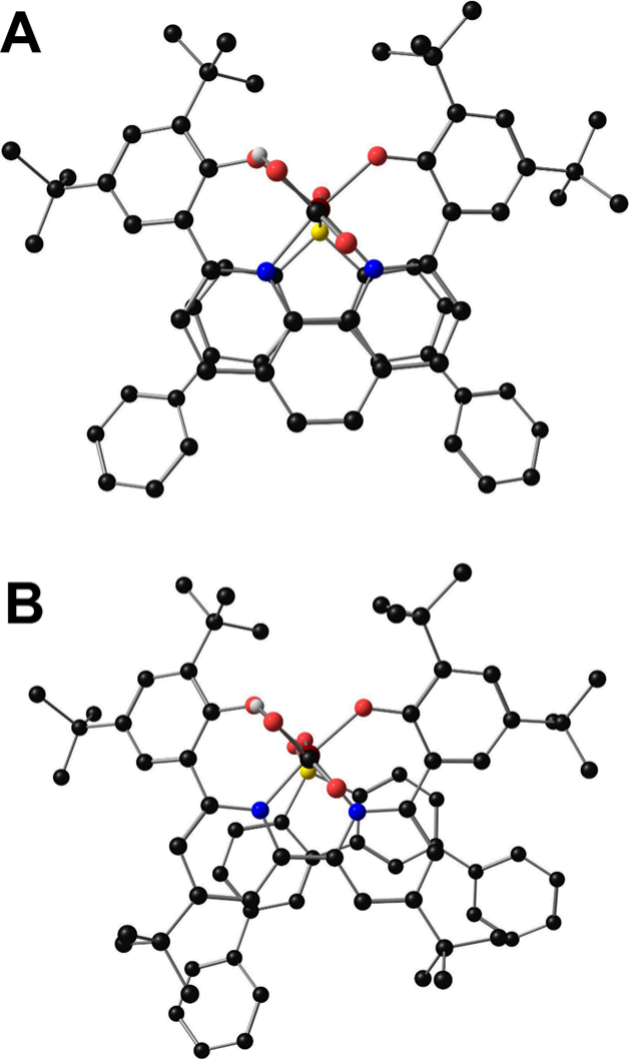
Molecular geometries for calculated structures of  where Cr is derived from the phen-based complex **3** (**A**) or the *tert*-butyl substituted bpy complex **4** (**B**) with select H atoms removed for clarity.

Based on these conclusions and hypotheses, coelectrocatalysis was also explored with the phen-based **3**, which has extended aromatic character relative to the bpy-based complexes.^37^ CPE experiments with CO_2_ saturation and added PhOH comparing the effect of including DBTD and Ph_2_DBTD with **3** again show that lower RM standard potentials result in greater TOF_CPE_ with Ph_2_DBTD (126 s^–1^) than DBTD (56.3 s^–1^). TOF_CPE_ values for **3** show greater enhancement than **1** with both RMs, even though the catalytically relevant reduction potentials of the two are within 10 mV. With Ph_2_DBTD as a RM, cocatalytic activity **3** increases by a factor of 26 compared to a factor of 10 for **1**, consistent with the proposal that the increased aromatic character of phen should improve PB between the RM and Cr compound. DFT methods estimated that the equilibrium binding reactions involving the radical ions of both DBTD and Ph_2_DBTD are ∼2 kcal/mol more favorable for **3** than **1**. However, the TOF_CPE_ values for **4** with either RM are still greater than **3**, most likely due to its higher intrinsic activity. With Ph_2_DBTD the cocatalytic activity increase for **4** was only by a factor of 21, suggesting that **4** is underperforming due to kinetic limitations on the association of the RM and Cr-complex. Indeed, DFT calculated structures show the impact of steric limitations on vertical atom–atom overlap between **4** and Ph_2_DBTD in the formation of the cocatalyst assembly *iv* (Figure 10). By comparison, **3** and Ph_2_DBTD have excellent vertical atom–atom overlap, indicative of a more ideal PB interaction.

To assess how the coordination strength of the RM might alter cocatalysis, 5-phenylbenzo[*b*]phosphinole-5-oxide (PhBPO) was selected as a RM to test with **1**, with the idea that a R_3_P=O moiety would form a stronger dative covalent interaction with Cr than R_2_S(O)=O.^39^ Like the sulfone-based RMs, PhBPO has a reversible one-electron reduction (*E*_1/2_ = −2.42 V vs Fc^+^/Fc) and does not bind with **1** under Ar saturation. The greater nucleophilicity of PhBPO is apparent under CO_2_ saturation, where control CVs showed a loss of reversibility and an increase in current suggestive of a slow chemical reaction with CO_2_ after reduction. Under CO_2_ saturation with added PhOH, however, an electrocatalytic CO_2_RR response is observed for **1** with PhBPO. CPE studies show the selective reduction of CO_2_ to CO with a TOF_CPE_ of 15 s^–1^ with **1** and PhBPO, which is less of an activity enhancement than is observed with DBTD as the RM. Computational and experimental results confirmed that the coordinating ability of reduced PhBPO is stronger than the reduced sulfone-based RMs and estimated a lower barrier for C–OH bond cleavage. Unfortunately, the corresponding beneficial impact on the TDTS barrier height does not result in improved TOFs. Since PB contributes relatively less to the interaction between the RM and Cr complex because of stronger inner-sphere binding, the ideal PB configuration becomes relatively less favorable. Overall, the PhBPO RM acts similarly to the sulfone-based RMs, suggesting that if the stronger axial bonding could be retained without interrupting vertical–vertical overlap between the RM and Cr complex, further activity enhancements are possible.

## Conclusions and Perspectives

Currently, we are aware of only one other Cr-based electrocatalyst system based on quaterpyridine with high selectivity for the CO_2_RR.^40^ Although initial rates are promising for the Cr(quaterpyridine) complex, the system lacks stability in the presence of the CO product and degrades after 30 min of electrolysis, unlike the systems presented here. Together with the mechanistic information presented above, it can be assumed that one of the keys to stable CO_2_RR with Cr metal centers is avoiding a formally Cr(0) oxidation state. Given the significant stability of Cr(CO)_6_, degradation is likely to be kinetically and thermodynamically facile, if the catalyst has any binding interaction with CO at reducing potentials. Thus, it can be reasonably concluded that some ligand charge is beneficial in avoiding Cr(0), especially in light of our results comparing bpy- and tpy-based ligand frameworks. Further, the antiferromagnetic pairing of a ligand-based radical with a high-spin Cr(II) center accesses a kinetic selectivity benefit that is diminished with further electron loading in ligand framework. This implies that single-electron redox activity is beneficial for avoiding protonation of the Cr center and HER.

Modification of the bpy-backbone results in activity scaling consistent with general observations in the field, where more negative reduction potentials produce greater TOFs.^38^ However, it is not yet apparent what alterations to the phenolate groups, either in terms of steric profile or electronic character, might enable in terms of activity tuning. For instance, the inclusion of pendent bases in this ligand framework has shown that secondary-sphere effects can be introduced to control kinetic selectivity in electrochemical reduction reactions,^41−43^ which may also be an effective tool for tuning the Cr-based CO_2_RR response. It is also possible that modification of the phenolates can be used to modify the *d* orbitals which participate in CO_2_ binding, given that they share symmetry with the relevant π-bonding manifold of the frontier orbitals.

The cocatalytic benefit of using a phen-based backbone implies that a further expansion of the redox activity and aromatic character of a diimine fragment in the ligand backbone could also be beneficial.^44,45^ More broadly, the cocatalytic studies make clear that future designs for inner-sphere RMs need to shift their redox potentials to overlapping with that of the Cr complex. The results of using a phosphole-based RM suggest that the simultaneous tuning of ligand donor properties should offer a pathway to further rate enhancements, if the ideal PB vertical atom–atom alignment can be conserved.

Overall, the systematic changes made to the Cr-based catalytic systems for the CO_2_RR have resulted in an increased understanding of how to design Cr-based electrocatalyst systems. Importantly, the results here offer multiple viable pathways for further improvement of the CO_2_RR with Cr-based complexes that are under ongoing investigation. Generally, these mechanistic lessons also have implications for the development of other small molecule transformations under electrocatalytic and coelectrocatalytic conditions, particularly with open-shell early transition metal active sites.^24^
